# Symptom prevalence, duration, and risk of hospital admission in individuals infected with SARS-CoV-2 during periods of omicron and delta variant dominance: a prospective observational study from the ZOE COVID Study

**DOI:** 10.1016/S0140-6736(22)00327-0

**Published:** 2022-04-23

**Authors:** Cristina Menni, Ana M Valdes, Lorenzo Polidori, Michela Antonelli, Satya Penamakuri, Ana Nogal, Panayiotis Louca, Anna May, Jane C Figueiredo, Christina Hu, Erika Molteni, Liane Canas, Marc F Österdahl, Marc Modat, Carole H Sudre, Ben Fox, Alexander Hammers, Jonathan Wolf, Joan Capdevila, Andrew T Chan, Sean P David, Claire J Steves, Sebastien Ourselin, Tim D Spector

**Affiliations:** a Department of Twin Research and Genetic Epidemiology, King's College London, London, UK; b School of Biomedical Engineering & Imaging Sciences, King's College London, London, UK; c Nottingham NIHR Biomedical Research Centre at the School of Medicine, University of Nottingham, Nottingham, UK; d ZOE, London, UK; e Department of Medicine, Samuel Oschin Comprehensive Cancer Institute, Cedars-Sinai Medical Center, Los Angeles CA, USA; f Department of Ageing and Health, Guy's and St Thomas' NHS Foundation Trust, London, UK; g MRC Unit for Lifelong Health and Ageing, University College London, London, UK; h Centre for Medical Image Computing, Department of Computer Science, University College London, London, UK; i King's College London & Guy's and St Thomas' PET Centre, London, UK; j Clinical and Translational Epidemiology Unit, Massachusetts General Hospital and Harvard Medical School, Boston, MA, USA; k NorthShore University Health System & University of Chicago Pritzker School of Medicine, Chicago, IL, USA

## Abstract

**Background:**

The SARS-CoV-2 variant of concern, omicron, appears to be less severe than delta. We aim to quantify the differences in symptom prevalence, risk of hospital admission, and symptom duration among the vaccinated population.

**Methods:**

In this prospective longitudinal observational study, we collected data from participants who were self-reporting test results and symptoms in the ZOE COVID app (previously known as the COVID Symptoms Study App). Eligible participants were aged 16–99 years, based in the UK, with a body-mass index between 15 and 55 kg/m^2^, had received at least two doses of any SARS-CoV-2 vaccine, were symptomatic, and logged a positive symptomatic PCR or lateral flow result for SARS-CoV-2 during the study period. The primary outcome was the likelihood of developing a given symptom (of the 32 monitored in the app) or hospital admission within 7 days before or after the positive test in participants infected during omicron prevalence compared with those infected during delta prevalence.

**Findings:**

Between June 1, 2021, and Jan 17, 2022, we identified 63 002 participants who tested positive for SARS-CoV-2 and reported symptoms in the ZOE app. These patients were matched 1:1 for age, sex, and vaccination dose, across two periods (June 1 to Nov 27, 2021, delta prevalent at >70%; n=4990, and Dec 20, 2021, to Jan 17, 2022, omicron prevalent at >70%; n=4990). Loss of smell was less common in participants infected during omicron prevalence than during delta prevalence (16·7% *vs* 52·7%, odds ratio [OR] 0·17; 95% CI 0·16–0·19, p<0·001). Sore throat was more common during omicron prevalence than during delta prevalence (70·5% *vs* 60·8%, 1·55; 1·43–1·69, p<0·001). There was a lower rate of hospital admission during omicron prevalence than during delta prevalence (1·9% *vs* 2·6%, OR 0·75; 95% CI 0·57–0·98, p=0·03).

**Interpretation:**

The prevalence of symptoms that characterise an omicron infection differs from those of the delta SARS-CoV-2 variant, apparently with less involvement of the lower respiratory tract and reduced probability of hospital admission. Our data indicate a shorter period of illness and potentially of infectiousness which should impact work–health policies and public health advice.

**Funding:**

Wellcome Trust, ZOE, National Institute for Health Research, Chronic Disease Research Foundation, National Institutes of Health, and Medical Research Council

## Introduction

On Nov 26, 2021, WHO designated the SARS-CoV-2 variant B.1.1.529 (omicron), first seen in South Africa, as a new variant of concern.^1^ Of the many mutations detected in omicron, more than 30 are in the spike protein and 15 are in the receptor-binding domain, which could affect transmission, disease presentation, and natural or vaccine-induced protective immunity.^1^ In the subsequent weeks, omicron spread to over 80 countries and became the dominant SARS-CoV-2 variant in the UK, overtaking the previously dominant delta variant (B.1.617.2) on Dec 20, 2021.^2^ Early reports suggested infection with omicron was less severe than with previous variants.^3^, ^4^, ^5^, ^6^ A small (n=40) South Korean study^4^ described the clinical presentation of omicron cases; there were no severe cases. Hospital admission rates in South Africa for cases infected with this variant have been significantly lower than for previous waves, in which other variants of concern were dominant.^5^, ^6^, ^7^, ^8^ Similarly, a study^9^ investigating the first 1119 omicron cases in France reported significantly lower rates of hospitalisation, need for intensive care, and mortality compared with 3075 delta cases.^9^ However, no detailed published reports have investigated symptom prevalence and acute symptom duration, and how these compare to the delta variant.

**Research in context**

**Evidence before this study**

We searched PubMed for articles published up to Jan 24, 2022, using the terms “SARS-CoV-2 omicron symptoms” and “SARS-CoV-2 omicron hospitalisation”. We found several published papers and preprints covering differences in viral load, neutralising antibody responses among vaccinated individuals, and the description of prevalence in various regions (eg, Florida, Norway, and South Africa). Several studies from South Africa indicated that infection with the omicron variant was significantly less severe than with the previous dominant variants, with lower rates of hospital admission. A cohort study at IHU Méditerranée Infection investigated the first 1119 omicron cases in France and reported significantly lower rates of hospitalisation, need for intensive care, or mortality compared with 3075 delta cases. A small study from Korea (n=40) showed that symptoms for omicron were mild and no patients needed supplemental oxygen. However, the general presentation of symptoms compared with delta and how the duration and risk of hospitalisation vary in patients who have received two or three vaccine doses has not been reported on a large prospective population scale.

**Added value of this study**

This is a larger, more detailed, generalisable, and less confounded study than attempted previously. We report that the symptoms characterising an omicron breakthrough infection differ from those of the delta SARS-CoV-2 variant. Since we matched on age, sex, and number of vaccine doses, these factors are unlikely to confound our observation.

**Implications of all the available evidence**

Our study substantiates previous suggestions that the omicron SARS-CoV-2 variant has a different clinical presentation to that of previous waves of COVID-19 in vaccinated individuals. However, this might not be the case in unvaccinated individuals. The different clinical presentation is important for selection of test-triggering symptoms. The shorter presentation of symptoms suggests (pending confirmation from viral load studies) that the period of infectiousness might be shorter, which would in turn impact workplace health policies and public health guidance.

We aimed to quantify the differences in symptoms, risk of hospital admission, and duration following infection with the omicron or delta variants among people vaccinated (two or three doses) in a large community cohort from the UK drawn from the ZOE COVID Study app.

## Methods

### Study design and participants

The study design and analytical pipeline to generate the study samples can be found in the appendix (p 4).

We collected prospective longitudinal observational data using the ZOE COVID Study, previously known as the COVID Symptoms Study App.^10^ The app enables self-reported information related to SARS-CoV-2 infection to be captured. Upon enrolment, users provided baseline demographic and health information. Subsequently, participants provided daily updates on symptoms experienced, SARS-CoV-2 test results, vaccines administered, and if they were self-quarantining or seeking health care, including the level of intervention and related outcomes. Users can also proxy-report for others. Ethical approval for use of the app for research purposes in the UK was obtained from King's College London Ethics Committee (LRS-19/20-18210), and all users provided consent for non-commercial use.

### Procedures

We included data from all UK participants aged 16–99 years (including proxy-reported individuals), with a body-mass index between 15 and 55 kg/m^2^, who had at least two doses of any SARS-CoV-2 vaccine, were symptomatic, and logged a positive symptomatic PCR or lateral flow antigen test (LFAT) for SARS-CoV-2 between June 1, 2021, and Jan 17, 2022. For more details on inclusion and exclusion criteria, see the appendix (pp 2, 4). The full list of symptoms queried is presented in the appendix (pp 6–7).

### Outcomes

Our primary outcomes were the likelihood of developing a given symptom (from the 32 monitored in the app) within 7 days before or after the positive LFAT or PCR in those infected in the omicron-dominant period compared with in those infected in the delta-dominant period, the likelihood of reporting any of the classic symptoms (fever, loss of smell, or persistent cough^10^), and the likelihood of hospital admission within the disease period in the same populations.

Our secondary outcome was symptoms duration for omicron versus delta. Acute symptom duration was calculated as the difference between the onset date (date of reported symptoms, unhealthy log: “I am not feeling quite right”) and recovery date (the first day where users reported feeling normal, healthy log: “I feel physically normal”, which was not followed by a day with reported symptoms for at least one week). For this secondary analysis, we included a subset of individuals who reported at least weekly from first symptom report until symptom-freedom and had recovered within 21 days, to allow us to include sufficient individuals.

### Statistical analysis

Statistical analysis was done using Python version 3.8.10 (pandas, NumPy, SciPy, statsmodel).

We compared data from two time periods: June 1, to Nov 27, 2021, when the delta variant was dominant in the UK (prevalence >70%); and Dec 20, 2021, to Jan 17, 2022, when omicron became dominant in the UK (prevalence >70%). Through a Euclidean distance-based algorithm,^11^ participants infected during omicron prevalence were matched 1:1 to participants infected during delta prevalence on age, sex, and vaccination doses. We were unable to match for SARS-CoV-2 prevalence, tiered lockdown restrictions, or vaccination rates, which varied widely across the community and over time during this study.

Baseline characteristics are presented as the number (%) for categorical variables and the mean (SD) for continuous variables.

We employed multivariable ordinary logistic regressions, as implemented in the Python package statsmodel (formula.api), to investigate the odds of developing symptoms and of having a severe outcome in participants infected during omicron prevalence compared with participants infected during delta prevalence, adjusting for age, sex, presence of comorbidities (cancer, diabetes, heart disease, lung disease, kidney disease, or use of immunosuppressants), vaccination status (2 *vs* 3 doses), and multiple testing (using Benjamini Hochberg correction, false discovery rate<0·005). We assessed risk of developing specific symptoms within 7 days either side of the positive test. Multivariable ordinary logistic regression adjusting for covariates was also employed to investigate whether symptom duration significantly differed between those infected during omicron prevalence versus those infected during delta prevalence. For this analysis, we compared the odds of symptoms lasting for over 7 days with the odds of symptoms lasting less than 7 days. See the appendix (p 3) for the script.

We did sensitivity analyses stratifying according to the number of vaccine doses to minimise the influence of confounding by vaccination status in the clinical presentation of the two variants. It was not possible to match for the same timespan since vaccination given the two different periods of dominance of the variants, nor could we run comparable matched analyses in the unvaccinated or in those who had received only one dose due to the small number of these individuals in the study population.

### Role of the funding source

The funders had no role in design or interpretation of the data. ZOE, funded by the Department of Health and Social Care, made the app available for data collection as a not-for-profit endeavour. Employees from ZOE carried out the statistical analyses. Representatives of ZOE approved the final manuscript for submission.

## Results

We included 63 002 app users who tested positive for SARS-CoV-2 by PCR or LFAT between June 1, 2021, and Jan 17, 2022; reported symptoms within the requisite timeframes; and logged at least weekly, from first symptom report until returning to symptom-freedom for calculation of illness duration. Of these, 33 785 users tested positive when delta was dominant, and 29 217 users tested positive when omicron was dominant. After 1:1 matching, the total number of participants in each of the two groups reduced to 4990. The demographic characteristics of the study population are presented in table 1.

**Table 1:** Participant demographics

		**Overall**		**1:1 matched sample**	
		Omicron	Delta	Omicron	Delta
Total (n)		29 217	33 785	4990	4990
Sex					
	Female	18 709 (64%)	21 296 (63%)	3302 (66%)	3302 (66%)
	Male	10 506 (36%)	12 489 (37%)	1688 (34%)	1688 (34%)
Age range, years		16–98	16–98	16–93	16–93
Mean (SD) age by number of vaccine doses, years					
	Two doses	40·35 (14·10)	52·40 (12·19)	40·35 (14·10)	40·58 (13·79)
	Three doses	54·84 (13·50)	59·29 (13·27)	59·29 (13·27)	59·29 (13·27)
BMI, kg/m^2^, mean (SD)		26·62 (5·41)	26·99 (5·55)	26·07 (5·47)	26·3 (5·73)
Health-care workers		1922 (7%)	1519 (4%)	256 (5%)	387 (8%)
Comorbidities		5309 (18%)	6292 (19%)	638 (13%)	967 (19%)
	Two vaccine doses	3929 (13%)	32 724 (97%)	3929 (79%)	3929 (79%)
	Three vaccine doses	25 288 (87%)	1061 (3%)	1061 (21%)	1061 (21%)
Number of distinct symptoms, median (IQR)		7 (5–11)	9 (6–13)	8 (5–12)	9 (6 −13)
Ethnicity					
	White	27 930 (96%)	32 540 (96%)	4699 (95%)	4771 (96%)
	Other	1287 (4%)	1245 (4%)	291 (5%)	219 (4%)
Recovered within 21 days		7139 (24%)	16 034 (47%)	1257 (25%)	1257 (25%)
Average symptom duration, days, mean (SD)		8·06 (4·84)	9·90 (5·14)	6·87 (5·21)	8·89 (5·04)

Data are n (%) unless otherwise specified. BMI=body-mass index. Patients with BMI less than 15 or over 55 were excluded from the analysis. Comorbidity numbers reflect the number of people presenting with at least one comorbidity (cancer, diabetes, heart disease, lung disease, kidney disease, or use of immunosuppressants).

**Figure 1:**
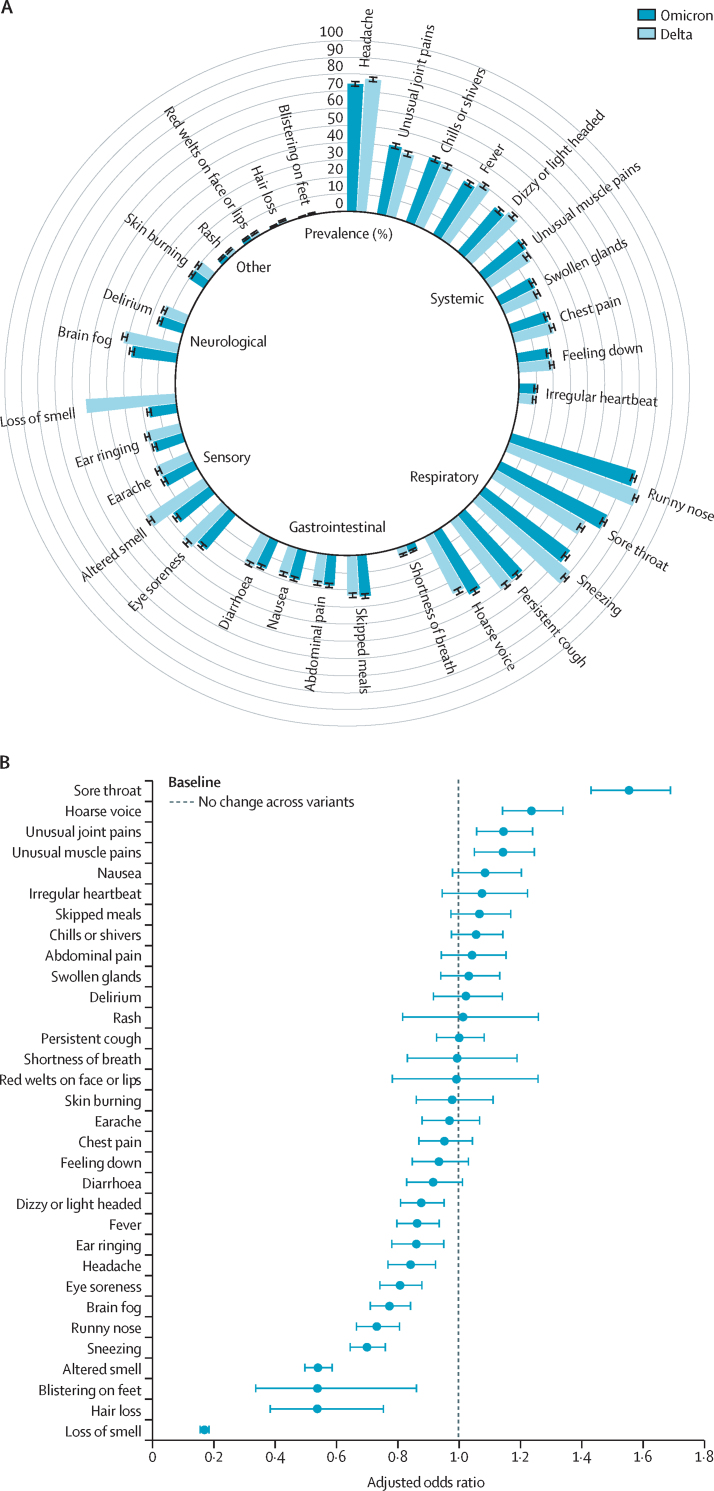
Symptoms in patients with delta or omicron SARS-CoV-2 infection (A) Prevalence of symptoms reported by individuals infected during omicron or delta prevalence. Data are percentage prevalence in the 1:1 matched sample. Error bars indicate 95% CI. (B) Association between symptoms and COVID-19 infection in 4990 participants who tested positive for SARS-CoV-2 when omicron was dominant and 4990 participants who tested positive when delta was dominant. Data are odds ratios comparing omicron and delta prevalence. Error bars indicate 95% CI.

Among participants reporting one or more potential symptoms of COVID-19 in the matched group and testing positive when delta was dominant, the most frequently reported symptoms from 4990 individuals were runny nose (4073 [81·6%]), headache (3888 [77·9%]), sneezing (3529 [70·7%]), sore throat (3033 [60·8%]), and loss of smell (2631 [52·7%]). Among those testing positive when omicron was dominant, the most frequently reported symptoms were runny nose (3818 [76·5%] of 4990 individuals), headache (3729 [74·7%]), sore throat (3517 [70·5%]), sneezing (3143 [63·0%]) persistent cough (2486 [49·8%]), and hoarse voice (2145 [42·6%], figure 1A). In the matched sample, the median number of symptoms was lower in the omicron prevalent group than in the delta prevalent group (table 1). The breakdown of symptoms prevalence in the 7 days before and after the positive test is depicted in figure 1A. Symptom prevalence in this 1:1 matched subset was consistent with overall prevalence in the larger set of 63 002 cases (appendix p 5), although we observed a slight discrepancy with respect to gastrointestinal symptoms.

**Figure 2:**
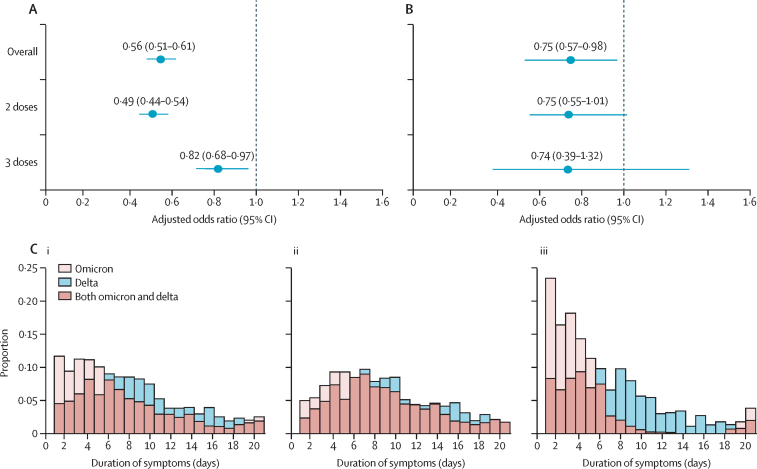
Classic symptoms, hospital admission, and symptom duration in patients infected with SARS-CoV-2 during delta and omicron prevalence (A) Association between type of SARS-CoV-2 prevalence (omicron or delta) and presentation of classic symptoms (defined as at least one of loss of smell, fever, or persistent cough) in 4990 participants who tested positive for SARS-CoV-2 when omicron was dominant and 4990 participants who tested positive when delta was dominant. Error bars indicate 95% CI. (B) Self-reported hospital admission by SARS-CoV-2 variant prevalence in the same subset as (A). (C) Acute symptom duration. Proportion of participants self-reporting symptoms to the ZOE COVID Study app within 21 days after infection with SARS-CoV-2 in (i) the overall matched set, (ii) participants who received two doses of any SARS-CoV-2 vaccine, and (iii) participants who received three doses of any SARS-CoV-2 vaccine.

We then assessed the difference in the prevalence of the various symptoms by deriving an odds ratio (OR) for omicron versus delta in the matched group (ie, the odds of a symptom being detected in participants infected during omicron prevalence compared with people infected during delta prevalence; figure 1B). Of the 32 symptoms assessed, 12 were significantly less prevalent (false discovery rate p<0·05) among individuals infected during omicron prevalence than among those infected during delta prevalence (loss of smell OR 0·17, 95% CI 0·16–0·19 [p<0·001]; altered sense of smell 0·54, 0·50–0·59 [p<0·001]; sneezing 0·70, 0·65–0·76 [p<0·001]; runny nose 0·73, 0·67–0·81 [p<0·001]; brain fog 0·78, 0·71–0·85 [p<0·001]; eye soreness 0·81, 0·74–0·88 [p<0·001]; headache 0·84, 0·77–0·93 [p<0·001]; fever 0·87, 0·80–0·94 [p<0·001]; hair loss 0·52, 0·37–0·75 [p<0·001]; blistering on feet 0·52, 0·31–0·86 [p=0·01]; ear ringing 0·86, 0·78–0·96 [p=0·005]; and dizzy or light headed 0·88, 0·81–0·96 [p=0·003]). However, sore throat and hoarse voice were significantly more likely to be present during omicron prevalence than during delta prevalence (sore throat OR 1·55, 95% CI 1·43–1·69 [p<0·001]; hoarse voice 1·24, 1·14–1·34 [p<0·001]). Participants infected during omicron prevalence were less likely to display at least one out of the three classic COVID-19 symptoms (fever, loss of smell, and persistent cough) compared with individuals infected with delta (OR 0·56, 95% CI 0·51–0·61; p<0·001, figure 2A).

We also did a sub-analysis assessing the ORs by vaccination status, which showed consistent results (appendix pp 8–11). We found that participants who had received two or three vaccine doses had a lower risk of hospitalisation during omicron prevalence (omicron 94 [1·9%] of 4990 individuals, delta 130 [2·6%] of 4990; OR 0·75, 95% CI 0·57–0·98; p=0·03, figure 2B). Given the small number of patients admitted to hospital in this community-based study, this effect did not reach statistical significance in the separate two-dose and three-dose groups, but the effects were similar in both (figure 2B).

In a subset of participants that recovered within 21 days, we compared the number of days spanned until resolution of acute symptoms in matched delta-infected and omicron-infected individuals (1530 in each group). We found that the duration of acute symptoms was longer for delta (mean duration 8·89 days, 95% CI 8·61–9·17; median duration 8·0 days, IQR 5·0–12·0) than for omicron (mean duration 6·87 days, 6·58–7·16; median duration 5·0 days, IQR 3·0–9·0; p<0·0001, figure 2C). This difference appeared less marked among individuals who had received only two doses of the vaccine (delta mean duration 9·57 days, 9·22–9·92; median duration 9·00 days, 6·00–13·00; omicron mean duration 8·30 days, 7·96–8·65; median duration 7·00, 4·00–11·00 days; p<0·0001, figure 2C) and more marked in individuals who had received three doses of the vaccine (delta mean duration 7·71 days, 7·26–8·15; median duration 7·00 days, 4·00–10·00; omicron mean duration 4·40 days 3·98–4·82, median duration 3·0 days, IQR 2·00–5·00; p<0·0001, figure 2C). Finally, in this same subset of individuals we investigated the odds of recovering within 7 days of the onset of symptoms during omicron prevalence compared with during delta prevalence. We obtained an OR (adjusted for age, sex, presence of comorbidities, and vaccine doses) of 2·49 (95% CI 2·10–2·95) indicating that people infected during omicron prevalence are twice as likely to recover within one week of the onset of symptoms than were those infected during delta prevalence.

## Discussion

The symptoms that characterise an omicron infection differ moderately from those of the delta SARS-CoV-2 variant. The two symptoms that were consistently more prevalent among omicron than among delta cases (regardless of vaccination status) were sore throat and hoarse voice. Four of the 32 symptoms assessed were significantly less prevalent during omicron prevalence than during delta prevalence in both vaccination groups (loss of smell, altered smell, eye soreness, and sneezing) and 12 out of the 32 symptoms were significantly less prevalent overall during omicron prevalence than during delta prevalence. The most striking difference was observed for loss of sense of smell, a pathognomonic feature of earlier waves of SARS-CoV-2 infection,^10^ now present in less than 20% of cases. Moreover, many debilitating symptoms such as brain fog, eye burning, dizziness, fever, and headaches were all significantly less prevalent in omicron cases.

Additionally, hospital admission was significantly lower in patients infected during omicron prevalence than in patients infected during delta prevalence. This supports previous findings from South Africa and South Korea,^4^, ^5^, ^6^, ^7^ which showed the omicron variant to be milder in terms of severity. Finally, the duration of acute symptoms was shorter during omicron prevalence than during delta prevalence, with the average presentation of omicron being 2 days shorter than that of delta. Furthermore, a third dose of vaccine was associated with a greater reduction in symptom duration in participants infected during omicron prevalence compared with those infected during delta prevalence.

SARS-CoV-2 is known to affect various organs in addition to the respiratory tract, leading to dermatological complications, myocardial dysfunction, gastrointestinal symptoms, neurological illnesses, hepatic injury, and renal injury.^12^, ^13^ Multiple organ involvement is particularly noticeable in severe cases.^14^ Our data indicate a narrower spectrum and faster resolution of symptoms with omicron, along with a milder presentation than with delta, all of which concur with the view that omicron seems to be much more transmissible than previous variants,^15^ but less severe in vaccinated populations.^16^ This is also consistent with in vitro studies^17^ that report that omicron replicates faster than all other SARS-CoV-2 variants in the bronchus but less efficiently in the lung parenchyma, and appears to enter human cells by a different route than other variants.^17^ Our finding that the odds of hospitalisation are 25% lower for omicron than for delta are consistent with reports by the South African private health insurer Discovery Health in Johannesburg,^16^ which announced that risk of hospital admission is 29% lower among people infected with omicron than those infected with delta.

The genome of SARS-CoV-2 omicron virus has some deletions and more than 30 mutations from the original sequence.^18^ Several mutations overlap with those present in the previous variants of concern and are known to increase transmissibility; other omicron mutations with known effects confer an increase in transmissibility and affect binding affinity.^19^ However, the effects of most of the remaining omicron mutations are not known.^20^ Characterising the differences in the clinical presentation of infection by omicron versus delta is not only of direct public health relevance as the public and clinicians are aware of what symptoms to look out for, but will also assist in understanding the potential effects of future variants of concern.

Our study has several strengths, including the one-to-one matched study design, whereby individuals infected during delta and omicron prevalence were matched for age, sex, and vaccination status; the community nature of the study; and the use of a mobile app for daily logging. This allowed us to assess the duration and risk of hospital admission and of acute symptoms in community cases logging prospectively, rather than a biased sample that would be derived retrospectively from a secondary health-care setting.

We also note some study limitations. First, we were unable to compare symptoms, risk of hospital admission, or duration of infection by the two variants in unvaccinated individuals, as most study participants were vaccinated. Also, hospital admission was not ascertained from surveillance systems, but was self-reported. Second, infection with omicron and delta were assigned based on the prevalence in the UK population at the time and not on individual sequencing from these individuals, and our use of self-reported data can introduce information bias. Although this factor might introduce some misclassification, over 70% of SARS-CoV-2 sequenced cases by TaqPath laboratories (by daily reports from the UK Health Security Agency^3^) were either delta or omicron according to the assigned period. Third, some participants might be more likely to report symptoms than others, and participants using the app were a self-selected group and not representative of the general population, although we have previously found that our self-reported data aligns well with surveys designed to be representative of the population.^21^, ^22^ Fourth, our populations were matched for age, vaccination status, and sex but not for any other potential confounders. This might also explain the slight discrepancies we observe in gastrointestinal symptoms between our overall sample and matched sample. Gastrointestinal symptoms queried could be influenced by a variety of lifestyle factors, none more so than dietary intake; by matching the sample, we could reduce some of this confounding lifestyle effect.^23^ Fifth, we were unable to assess the role of previous infection on clinical presentation due to insufficient sample size. Sixth, our data are limited by the initial UK vaccine roll-out's focus on health-care workers, older people, and the clinically vulnerable.^24^ In addition, symptoms present at the time of infection might be related to viral or bacterial co-infections at the time of the SARS-CoV-2 infection. Finally, although the study design was matched for vaccination status (two or three doses), we could not match for time elapsed since vaccination. However, this would probably bias the data in terms of presenting a more severe clinical picture for omicron than if individuals had been matched for time since vaccination,^25^ and we believe that this would strengthen the robustness of our key finding, that omicron has a less severe clinical presentation than delta.

Using a matched design we report that among vaccinated individuals, the clinical symptoms associated with symptomatic infection by the SARS-CoV-2 omicron variant are different, milder, and of shorter duration than those presented by the delta variant among vaccinated individuals. Furthermore, loss of smell, so central in the clinical presentation of COVID-19, is much less frequently reported by those infected with omicron.

## Data sharing

Anonymised research data are shared with third parties via the centre for Health Data Research UK (HDRUK.ac.uk). US investigators are encouraged to coordinate data requests through the Coronavirus Pandemic Epidemiology (COPE) consortium (www.monganinstitute.org/cope-consortium). Data updates can be found on https://covid.joinzoe.com

## Declaration of interests

TDS, AMV, CJS, and SO are consultants to ZOE. JW, AM, LP, SP, and JC are employees of ZOE. All other authors declare no competing interests.
