# Supplementary material for: Symptom prevalence, duration, and risk of hospital admission in individuals infected with SARS-CoV-2 during periods of omicron and delta variant dominance: a prospective observational study from the ZOE COVID Study
Source: Lancet. 2022 Apr 23;399(10335):1618–24. doi: 10.1016/S0140-6736(22)00327-0 (PMC8989396; doi:10.1016/S0140-6736(22)00327-0)
Supplement: Supplementary appendix [file mmc1.pdf]

THE LANCET

Supplementary appendix

This appendix formed part of the original submission and has been peer reviewed. We post it as supplied by the authors.

Supplement to: Menni C, aldes A M, Polidori L, et al. Symptom prevalence, duration, and risk of hospital admission in individuals infected with SARS-CoV-2 during periods of omicron and delta variant dominance: a prospective observational study from the ZOE COVID Study. *Lancet* 2022; published online April 7. https://doi.org/10.1016/S0140-6736(22)00327-0.

A comparison of SARS-CoV-2 symptom prevalence, duration and risk of hospitalisation among vaccinated individuals from the ZOE COVID Study during Omicron versus Delta variant dominant periods

Cristina Menni[1*], PhD, Prof. Ana M. Valdes[2,*], PhD, Lorenzo Polidori[3], MSc, Michela Antonelli[4], PhD, Satya Penamakuri[3], MSc, Ana Nogal[1], MSc, Panayiotis Louca[1], MSc, Anna May[3], MA, Jane C. Figueiredo[5], PhD, Christina Hu[3], MA, Erika Molteni[4], PhD, Liane Canas[4], PhD, Marc F Österdahl[1,6], MBBS, Marc Modat[4], PhD, Carole H. Sudre[4,7,8], PhD, Ben Fox[3], BSc, Prof. Alexander Hammers[4,9], PhD, Jonathan Wolf[3], MA, Joan Capdevila[3], PhD, Prof. Andrew T Chan, M.D[10], Prof. Sean P David[11], MD, Claire J Steves[1,6], PhD, Prof. Sebastien Ourselin[4§], PhD, Prof. Tim D. Spector[1*§], MD

APPENDIX

Table of Contents

Supplementary Methods 2
Exclusion Criteria 2
Statistical analysis main script 2

Figure S1. Consort diagram of study design and analytical pipeline. 4

Figure S2. Prevalence of symptoms reported by individuals infected when the Omicron variant (navy) or the Delta variant (blue) was dominant. 5

Table S1. List of symptoms queried**.** 6

Table S2. Association between symptoms and type of COVID-19 (Omicron or Delta) infection in 2x 4,990 participants testing positive for SARS-CoV-2 by vaccination status. 8

Supplementary Methods

Exclusion Criteria

We excluded participant reporting symptoms within 7 days after vaccination because symptoms in that period may not be related to the infection but reflect reactions to the vaccine.

Individuals under the age of 16 were excluded as vaccination in the UK for this age group only started in September 2022 and second doses for this group have only become available from February 10th, 2022[1]. We further excluded those with a BMI > 55 kg/m2. Although there were very few outliers with extreme BMI values, such values are more likely to arise from incorrect data entry by the app user.

References

1 JCVI statement on COVID-19 vaccination of children aged 12 to 15 years: 3 September 2021. GOV.UK. https://www.gov.uk/government/publications/jcvi-statement-september-2021-covid-19-vaccination-of-children-aged-12-to-15-years/jcvi-statement-on-covid-19-vaccination-of-children-aged-12-to-15-years-3-september-2021 (accessed Feb 12, 2022).

Statistical analysis main script

```
# import the packages
import statsmodels.formula.api as smf
import statsmodels.api as sm
# fix the random seed for fitting
np.random.seed(seed=42)
# define the variables to include
EXOG = [‘is_omicron’, ‘age’, ‘gender’, ‘comor’, ‘vacc_status’]
#target = depending on the regression output we choose the target
# define the model to fit
model = smf.glm(
formula=f'{target} ~ C(is_omicron, Treatment(reference=0)) + age + comor + gender + vacc_status',
data=matched_group[EXOG], family = sm.families.Binomial())
        res=model.fit()
```

In this code, matched_group indicates the DataFrame including the matched participants on age, sex and vaccination status. The target variable of the model is a symptom when we compute ORs for symptom prevalences and it is hospitalisation (binary) when we compute its OR.

Figure S1. Consort diagram of study design and analytical pipeline.

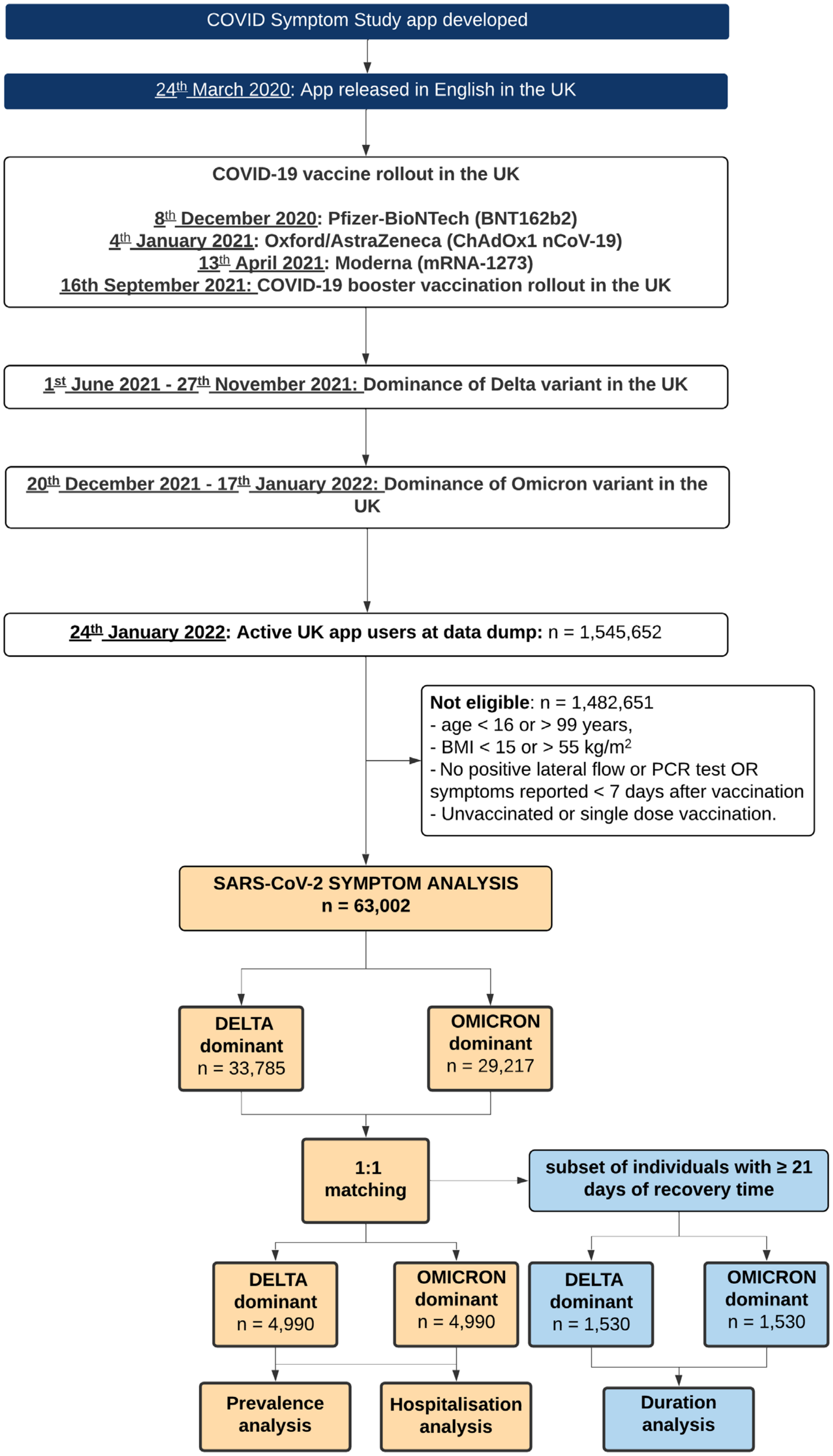

Figure S2. Prevalence of symptoms reported by individuals infected when the Omicron variant (navy) or the Delta variant (blue) was dominant. Average % and 95% CI in the overall sample are presented.

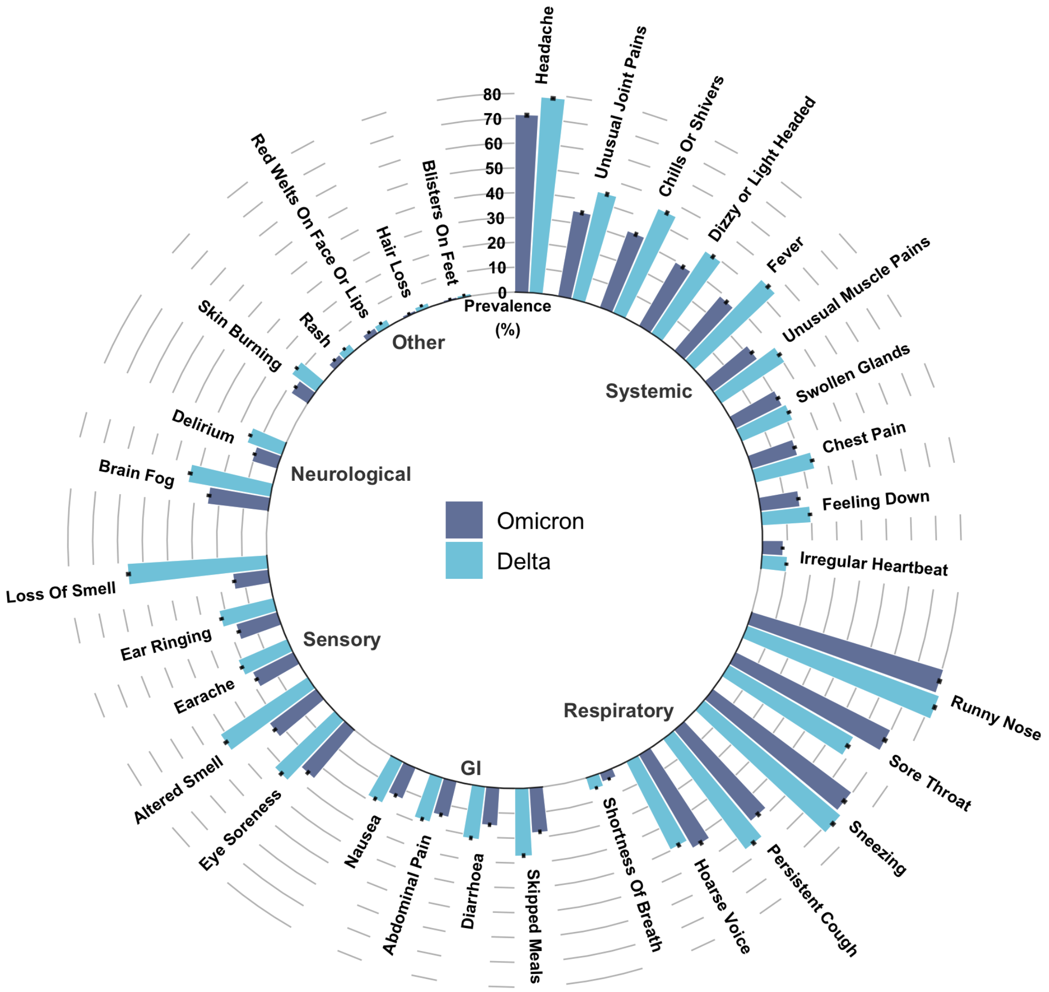

Table S1. List of symptoms queried. Mapping between symptom questions in the App and symptom names reported in the manuscript is presented.

Screen 1 - Are you experiencing any of the below symptoms?		Screen 2 - Are you experiencing any symptoms in your head area?		Screen 3 - Are you experiencing any symptoms in your throat or chest area?		Screen 4 - Are you experiencing any symptoms in your gut or stomach area?	
**Question in the App**	**Symptom name in the manuscript**	**Question in the App**	**Symptom name in the manuscript**	**Question in the App**	**Symptom name in the manuscript**	**Question in the App**	**Symptom name in the manuscript**
Fever of feel too hot	Fever	Headache	Headache	Sore or painful throat	Sore Throat	Unusual abdominal pain	Abdominal Pain
Chills or shivers (feel too cold)	Chills Or Shivers	Dizziness or light-headedness	Dizzy Light Headed	Swollen neck glands	Swollen Glands	Nausea or vomiting	Nausea
Unusual fatigue	Fatigue	Loss of smell / taste	Loss Of Smell	Unusually hoarse voice	Hoarse Voice	Diarrhoea	Diarrhoea
Rash on your arms or torso	Rash	Altered smell / taste (things smell or taste different to usual)	Altered Smell	Persistent cough (coughing a lot for more than an hour or 3 or more coughing episodes in 24 hours	Persistent Cough	Skipping meals	Skipped Meals
Red/purple sores or blisters on your feet, including your toes	Blisters On Feet	Runny nose	Runny Nose	Shortness of breath or trouble breathing	Shortness Of Breath		
Raised, red, itchy welts on the skin or sudden swelling of the face or lips	Red Welts On Face Or Lips	Sneezing more than usual	Sneezing	Unusual chest pain or tightness in your chest	Chest Pain		
Strange, unpleasant sensations in your skin like pins & needles or burning	Skin Burning	Unusual eye-soreness or discomfort (light sensitivity, excessive tears, or pink/red eye)	Eye Soreness	Usually fast or irregular heartbeat (palpitations)	Irregular Heartbeat		
Unusual hair loss	Hair Loss	Earache	Earache				

Unusual strong muscle pain or aches	Unusual Muscle Pains	Ringing in your ears	Ear Ringing				
Unusual joint paints or aches	Unusual Joint Pains						
Feeling down, depressed or hopeless	Feeling Down						
Loss of concentrating or memory (brain fog)	Brain Fog						
Confusion, disorientation or drowsiness	Delirium						

Table S2. Association between symptoms and type of COVID-19 (Omicron or Delta) infection in 2x4,990 participants testing positive for SARS-CoV-2 by vaccination status. Symptoms' prevalence and odds ratio comparing Omicron vs Delta are presented.

Symptom	Prevalence Delta (%)	Prevalence Omicron (%)	Vaccine status	OR [95%CI]
Abdominal Pain	18.5	13.9	2	1.09 [0.97 - 1.22]
Abdominal Pain	15.8	19.7	3	0.87 [0.69 - 1.11]
Altered Smell	41.9	22.3	2	0.53 [0.48 - 0.58]
Altered Smell	32.4	27.7	3	0.6 [0.49 - 0.73]
Blisters On Feet	1.0	0.5	2	0.48 [0.28 - 0.84]
Blisters On Feet	1.0	0.8	3	0.74 [0.3 - 1.87]
Brain Fog	33.6	24.2	2	0.76 [0.69 - 0.84]
Brain Fog	28.4	27.6	3	0.85 [0.69 - 1.03]
Chest Pain	25.8	24.7	2	0.96 [0.86 - 1.06]
Chest Pain	18.9	17.4	3	0.94 [0.75 - 1.18]
Chills Or Shivers	40.4	43	2	1.12 [1.03 - 1.23]
Chills Or Shivers	35.5	30.8	3	0.83 [0.69 - 0.99]
Delirium	16.0	16.1	2	1.01 [0.89 - 1.14]
Delirium	9.8	10.3	3	1.12 [0.84 - 1.49]
Diarrhoea	20.7	16.2	2	0.89 [0.8 - 1]

Symptom	Prevalence Delta (%)	Prevalence Omicron (%)	Vaccine status	OR [95%CI]
Diarrhoea	16.2	18.7	3	1.05 [0.83 - 1.32]
Dizzy Light Headed	39.6	35.8	2	0.86 [0.78 - 0.94]
Dizzy Light Headed	29.8	28.7	3	0.99 [0.82 - 1.2]
Ear Ringing	20.7	17	2	0.87 [0.78 - 0.98]
Ear Ringing	20.0	18.4	3	0.82 [0.66 - 1.03]
Earache	23.1	21.6	2	0.92 [0.83 - 1.03]
Earache	15.0	17.3	3	1.23 [0.97 - 1.56]
Eye Soreness	32.1	27.7	2	0.82 [0.74 - 0.9]
Eye Soreness	30.6	24.8	3	0.78 [0.64 - 0.95]
Feeling Down	21.1	20.4	2	0.96 [0.86 - 1.07]
Feeling Down	17.2	14.6	3	0.84 [0.67 - 1.07]
Fever	44.1	26.7	2	0.82 [0.75 - 0.9]
Fever	25.8	39.2	3	1.09 [0.9 - 1.33]
Hair Loss	2.1	0.9	2	0.51 [0.35 - 0.74]
Hair Loss	1.4	1.1	3	0.75 [0.33 - 1.69]
Headache	79.7	68.2	2	0.84 [0.75 - 0.93]
Headache	71.3	76.5	3	0.87 [0.72 - 1.06]

Symptom	Prevalence Delta (%)	Prevalence Omicron (%)	Vaccine status	OR [95%CI]
Hoarse Voice	39.6	42.8	2	1.15 [1.05 - 1.26]
Hoarse Voice	31.1	42	3	1.62 [1.35 - 1.94]
Irregular Heartbeat	10.5	11.3	2	1.1 [0.95 - 1.27]
Irregular Heartbeat	8.0	7.6	3	0.98 [0.71 - 1.35]
Loss Of Smell	56.7	17.6	2	0.16 [0.14 - 0.18]
Loss Of Smell	38	13.2	3	0.24 [0.2 - 0.3]
Nausea	13.8	12.6	2	1.12 [1 - 1.25]
Nausea	18.2	19.7	3	0.95 [0.73 - 1.23]
Persistent Cough	52.3	49.9	2	0.92 [0.84 - 1]
Persistent Cough	42	49.5	3	1.4 [1.18 - 1.67]
Rash	3.7	3.6	2	0.96 [0.75 - 1.21]
Rash	2	2.8	3	1.42 [0.8 - 2.51]
Red Welts On Face Or Lips	2	2.2	2	0.97 [0.75 - 1.26]
Red Welts On Face Or Lips	3.1	3	3	1.1 [0.6 - 2.01]
Runny Nose	80.9	82.6	2	0.66 [0.59 - 0.74]
Runny Nose	81.8	74.9	3	1.11 [0.89 - 1.39]
Shortness Of Breath	5.7	5.1	2	0.95 [0.78 - 1.15]

Symptom	Prevalence Delta (%)	Prevalence Omicron (%)	Vaccine status	OR [95%CI]
Shortness Of Breath	4	4.3	3	1.27 [0.82 - 1.97]
Skin Burning	8.6	8.1	2	0.98 [0.85 - 1.13]
Skin Burning	11.1	10.8	3	1.01 [0.74 - 1.39]
Skipped Meals	16.9	16.2	2	1.08 [0.98 - 1.2]
Skipped Meals	25.7	27	3	0.99 [0.79 - 1.25]
Sneezing	69.8	61.3	2	0.69 [0.62 - 0.75]
Sneezing	74.2	69.3	3	0.77 [0.63 - 0.93]
Sore Throat	63.4	71	2	1.42 [1.29 - 1.57]
Sore Throat	51	68.4	3	2.11 [1.76 - 2.53]
Swollen Glands	18.4	19	2	1.03 [0.93 - 1.14]
Swollen Glands	23.9	24.4	3	1.06 [0.85 - 1.33]
Unusual Joint Pains	40	43.5	2	1.17 [1.07 - 1.28]
Unusual Joint Pains	32.6	33	3	1.06 [0.88 - 1.27]
Unusual Muscle Pains	30.7	34.2	2	1.19 [1.08 - 1.31]
Unusual Muscle Pains	20.5	19.1	3	0.95 [0.76 - 1.18]
